# Editorial: Metabolic traits associated with neurodevelopmental and neuropsychiatric disorders

**DOI:** 10.3389/fgene.2023.1319341

**Published:** 2023-11-06

**Authors:** Rekha Jagadapillai, Katyayani Singh

**Affiliations:** ^1^ Department of Pediatrics, Pediatric Research Institute, University of Louisville, Louisville, KY, United States; ^2^ Department of Physiology, Institute of Biomedicine and Translational Medicine, University of Tartu, Tartu, Estonia; ^3^ Centre of Excellence in Genomics and Translational Medicine, University of Tartu, Tartu, Estonia

**Keywords:** metabolic traits, neuropsychiatric disorders, neurodevelopmental disorders, cardiovascular diseases, diabetes, Alzheimer’s disease

Specific metabolic traits or phenotypes determine the distinct cellular and biochemical features of the nervous system during embryonic development and later in adulthood. Metabolic syndrome (MetS) due to altered nutrient availability can disrupt brain development and aging, underlying a wide range of clinical manifestations that include psychiatric and neurological disorders. Patients with neurological and psychiatric disorders are predisposed to poor physical health, especially cardiovascular disease, diabetes, and obesity, and also carry a high risk of premature mortality. The cause of these adverse outcomes is complicated, as it can be factored in by lifestyle, socio-economic status, and environmental factors, with genetics also playing a role. It is important to explore and understand whether there is a possible connection between neuropsychiatric conditions and metabolic traits, which is the aim of this Research Topic. The main, intriguing questions are why and how the shift in metabolic traits is susceptible in some patients, while others may already pose a vulnerability toward developing these devastating conditions. This Research Topic features four original research articles that broaden our understanding of the metabolic hypothesis in the context of depression, schizophrenia, maternal Alzheimer’s disease, and dementia.

Khani et al. aimed to investigate the evidence for schizophrenia and its possible relation with cardiometabolic traits by applying a two-sample bi-directional Mendelian randomization analysis using publicly available large-scale genomic summary data. They found that schizophrenia was correlated with slightly higher low-density lipoprotein (LDL) and total cholesterol levels. However, the authors demonstrated that the level of these correlations were trivial and did not persist multiple testing corrections, suggesting that the cardiometabolic changes in patients are not solely related to schizophrenia itself. Instead, metabolic disorders in patients may be related to factors such as lifestyle, diet, side effects of antipsychotic drugs, and inflammatory responses. Genetics also plays an important role in the involvement of risk factors for metabolic diseases in schizophrenia patients.

Schizophrenia is often associated with neurodevelopmental disorders, as developmental abnormalities during the embryonic stage result in structural and functional impairments in the brain. Genetic studies have indicated the rs1635 variant of the NKALP (NFKB activating protein-like) gene as a risk factor for developing schizophrenia with a proposed role in neurodevelopment, immune response, and cognitive disorders. Yang et al. used a combination of techniques ranging from cognitive testing of patients with early-onset schizophrenia (EOS), adult-onset schizophrenia (AOS), peripheral blood analysis, and *in vivo* molecular experiments to understand the possible mechanism of risk variant rs1635 in neurodevelopment processes associated with cognition in schizophrenia patients. It was demonstrated that SNP rs1635 influences cognitive processing only in EOS patients and not in AOS. Nkapl functions in neuronal migration have been studied using *in utero* electroporation with specific EGFP plasmids in Nkapl^fl/fl^ embryonic brains. The results show abnormal embryonic radial migration, indicating a modulation of neocortical development upon Nkapl deletion. Reduced expression and a shift in the phosphorylation level of NKAPL-T152N were observed in schizophrenia patients. Taken together, the study suggests the implications of NKAPL-T152N on aberrant embryonic neuronal development, which affects cognitive impairments in EOS through NAKPL phosphorylation.

Non-alcoholic fatty liver disease (NAFLD) is an important health concern that is a risk factor for a range of metabolic diseases, such as cardiovascular risk and higher rates of chronic kidney disease; it can also develop into hepatocellular carcinoma. Studies have reported that the severity of NAFLD is linked with depression, as it adversely affects the management of chronic diseases. Manusev et al. examined the role of genetics and environment in the Mexican-American population of South Texas and provided evidence for the interaction of genetic factors with depression to stimulate the expression of hepatic fibrosis. The study describes a large percentage of the Mexican-American population of South Texas that faces inexplicably high rates of obesity, diabetes, and depression. Also underlined is the increasing occurrence of NAFLD worldwide.The prevalence of NAFLD in this South Texas community sheds light on the global increase in NAFLD. The authors assessed depression using the Beck Depression Inventory–II and found significant genetic and environmental associations for hepatic fibrosis and depression. Their future investigations will be directed toward finding the specific genes involved and their interactions.

Gestational diabetes mellitus (GDM) refers to insulin resistance that occurs in pregnant women. GDM is an increasing medical complication of pregnancy around the world, as it is associated with both maternal and neonatal adverse outcomes. Women with GDM are at high risk of developing Type-2 Diabetes after delivery. Reports signal an intricate association between GDM, heart disease (HD), high body mass index (BMI), Alzheimer’s disease (AD), and dementia, although the majority of these reports are observational studies. Due to the heterogeneity in the statistical results and inconsistent design, conflicting results make observational studies controversial. Sheng et al. used Mendelian Randomization (MR) and multivariable MR (MVMR) to control the bias estimation and methodically estimate the direct effect of GDM, HD, and high BMI on maternal AD and dementia. They found no significant causal relationship between GDM, HD, or high BMI on maternal AD and dementia using MR and MVMR analyses. The authors emphasize the importance of future investigations to understand the potential risks deriving from elevated levels of GDM, HD, or high BMI in maternal AD/dementia.

In summary, the articles on this Research Topic recap the association and the impact of metabolism on neurodevelopmental and neuropsychiatric disorders. It is important to understand whether there is cross-talk between neuropsychiatric and neurodevelopmental disorders and metabolic disorders to develop more effective treatment strategies. Additionally, we would like to thank all the authors and reviewers who contributed to this Research Topic, and we hope that these articles will help a vast scientific community achieve more research outcomes in this area.

